# Supplementary material for: The NMR structure of the Ea22 lysogenic developmental protein from lambda bacteriophage
Source: Sci Rep. 2024 Feb 1;14:2685. doi: 10.1038/s41598-024-52996-3 (PMC10834534; doi:10.1038/s41598-024-52996-3)
Supplement: Supplementary file 1 — Supplementary Information. [file 41598_2024_52996_MOESM1_ESM.pdf]

Supplementary Information

The NMR structure of the Ea22 lysogenic developmental protein from bacteriophage lambda

Cameron Goddard[1], Bożena Nejman-Faleńczyk[2], and Logan W Donaldson[1*]

[1]York University, Department of Biology, Toronto, ON, M3J1P3, Canada
[2]University of Gdańsk, Department of Molecular Biology, Gdańsk, 80-308, Poland
*e-mail: logand@yorku.ca

Table S1. Statistics for the λ Ea22 CTD ensemble of structures

NOE distance restraints in the ensemble[a]	1018
intraresidue	493
short ($\|i-j\| = 1$)	182
medium ($1 \le \|i-j\| \le 5$)	93
long ($\|i-j\| > 5$)	225
interchain	25
Hydrogen bond distance restraints	
HN–O / N–O pairs	23
Torsion angle restraints	
backbone (Φ / Ψ)	43
Structural quality analysis	
close contacts	25
RMS deviation of bond angles (deg)	0.4
RMS deviation of bond lengths (Å)	0.009
RMS deviation to the mean coordinates[b]	
all backbone / heavy atoms (Å)	1.3 / 1.9
ordered backbone / heavy atoms (Å)	0.6 / 1.0
Ramachandran plot[c] (%)	
residues in most favored regions	96.1
residues in additional allowed regions	3.8
residues in generously allowed regions	0.1
residues in disallowed regions	0.0

[a] None of the twenty structures in the ensemble (PDB: 8DSX) has a distance violation > 0.2 Å and a dihedral angle violation > 5°.

[b] Ordered residues (113-118, 121-126, 130-151, 160-171) are defined by a dihedral angle order parameter with S(Φ)+S(Ψ)>=1.8 as determined by PSVS.

[c] Determined by PROCHECK for all residues.

Table S2 — Summary of restraint violations for the ensemble of structures, sorted by CYANA target value. The last column lists NOE violations observed > 0.5 Å and the range of the violation in the ensemble). The torsion angle violations are attributed to the last two amino acids in structure (ϕ,ψ of His76 and ϕ of Met77). Residue numbering follows the expressed protein with affinity tags.

Structure	target function	distance upper limits (cutoff 0.01Å)			torsion angles (cutoff 5˚)			NOE violations (max>0.5 Å)
#	value	# violations	RMS (A)	max (A)	# violations	RMS (˚)	max (˚)	Chain1_Atom1-Chain2_Atom2 [restraint (Å); range (Å)]
1	9.64	0	0.000	0.00	2	0.594	5.5	
2	9.85	1	0.001	0.07	0	0.000	0.0	
3	10.30	1	0.009	0.49	0	0.000	0.0	A_Glu38HB-A_Leu75HD2 [4.45; 0.05-0.49]
4	10.62	0	0.000	0.00	0	0.000	0.0	
5	11.02	1	0.003	0.17	2	0.661	6.1	
6	11.36	1	0.009	0.49	0	0.000	0.0	A_Glu38HB-A_Leu75HD2 [4.45; 0.05-0.49]
7	11.40	5	0.011	0.53	0	0.000	0.0	A_Val21HG1-A_Lys31HB [3.95; 0.03-0.53]
8	11.89	0	0.000	0.00	0	0.404	3.7	
9	12.10	1	0.003	0.14	2	0.628	5.8	
10	12.29	0	0.000	0.00	0	0.300	2.8	
11	12.36	3	0.013	0.66	2	0.721	6.7	A_Phe18HD1-A_Ile56HD1 [4.05; 0.03-0.66]
12	12.95	1	0.000	0.01	2	1.688	15.6	
13	13.02	2	0.021	1.19	0	0.000	0.0	A_Val29HG1-B_Val63HB [4.95; 0.07-1.19]
14	13.04	3	0.005	0.22	2	2.586	23.8	
15	14.60	0	0.000	0.00	0	0.018	0.2	
16	14.68	2	0.001	0.06	4	4.181	36.3	
17	14.89	2	0.001	0.06	2	2.671	24.7	
18	15.65	1	0.002	0.10	0	0.384	3.3	
19	18.09	1	0.013	0.72	4	3.969	36.1	A_Leu37HD1-A_Met78_HE [5.80; 0.04-0.72]
20	21.97	3	0.003	0.15	6	6.093	51.8	
Average	13.09	1	0.005	0.25	1	1.245	11.1	
+/-	2.90	1	0.006	0.32	2	1.705	14.9	

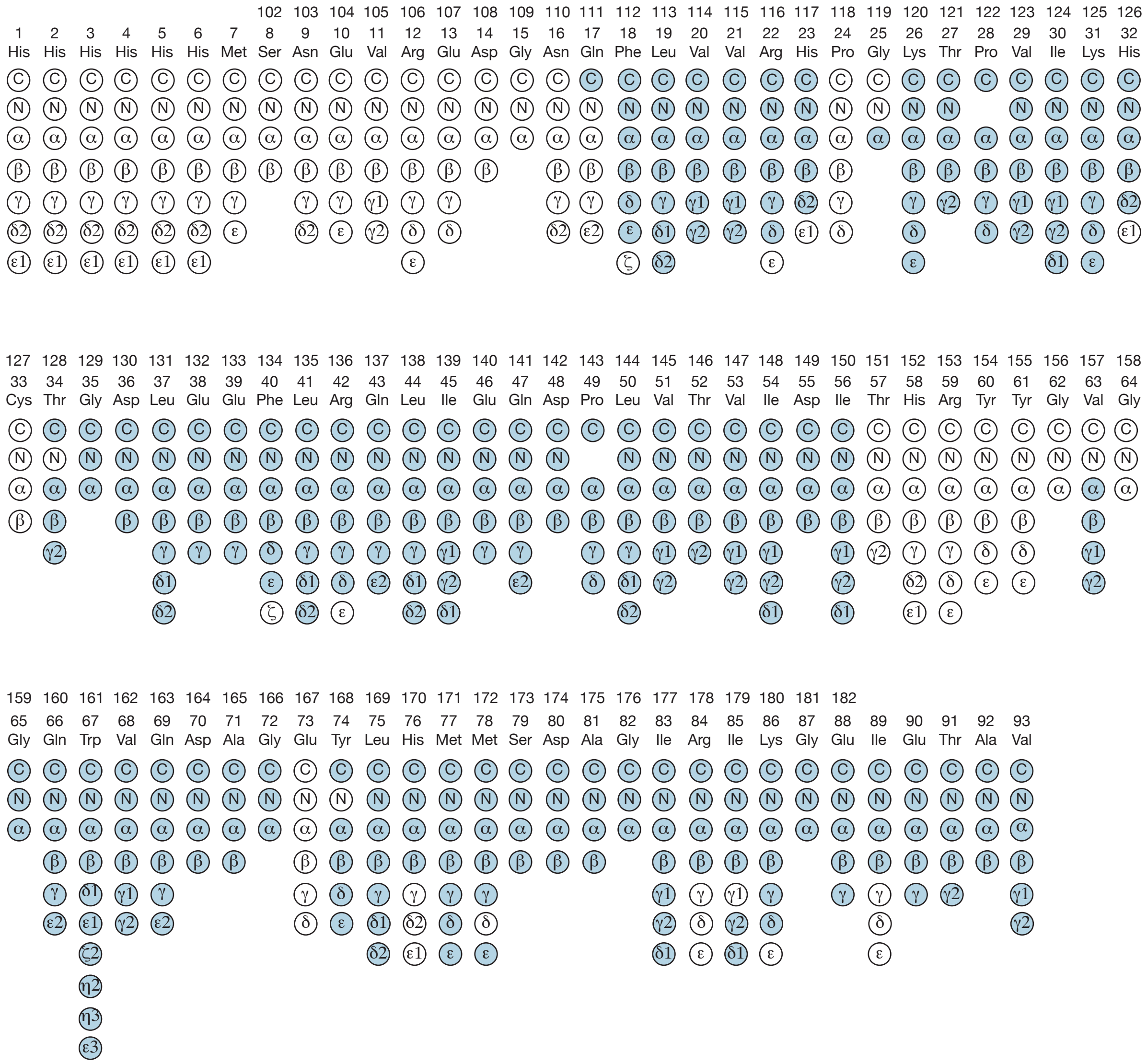

Figure S1 — Heavy atom chemical shift assignment graph for Ea22 as expressed (clear, unassigned; shaded, assigned). The native numbering of Ea22 is shown above the expressed protein numbering.

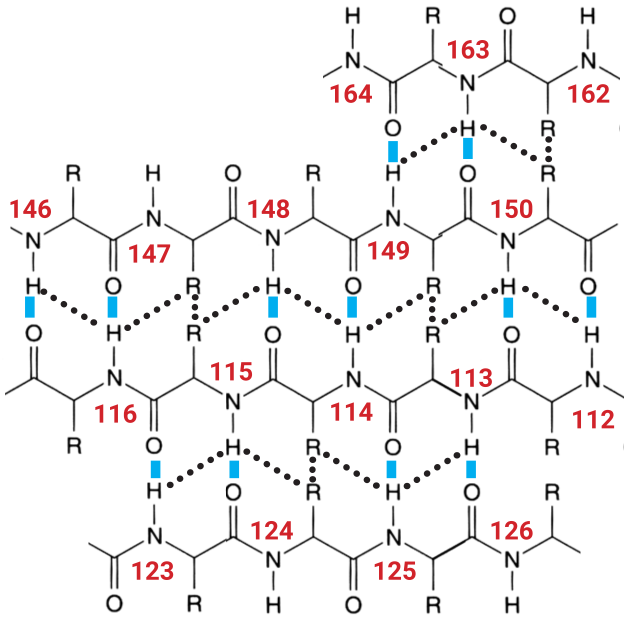

Figure S2 — An anti-parallel β-sheet identifed in the Ea22 C-terminal domain monomer by backbone NOEs observed in ^{13}C- and ^{15}N-edited NOESY spectra.

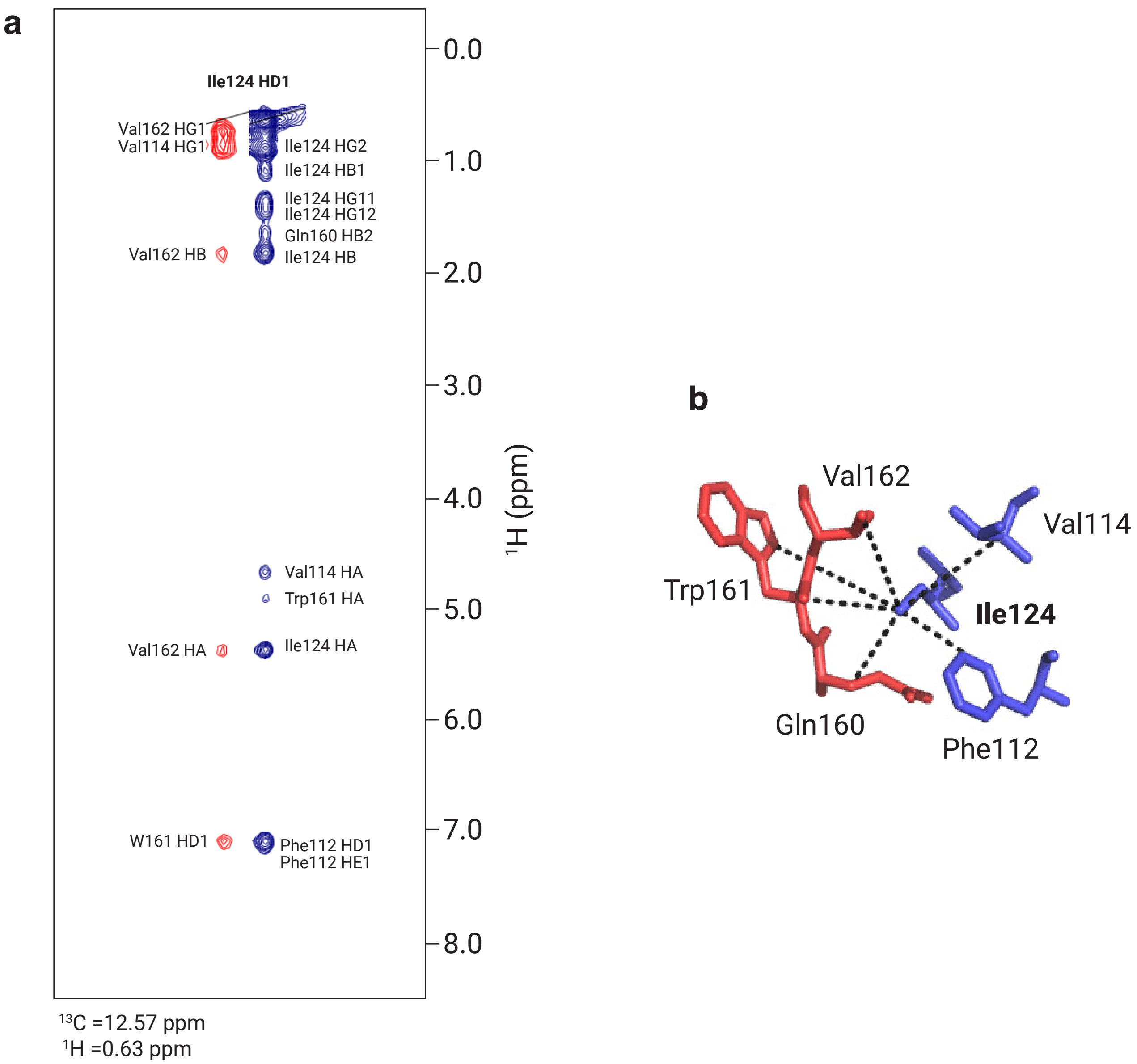

Figure S3 — Intramolecular and intermolecular observations supporting the structure of the Ea22 C-terminal domain. **(a)** A representative plane from a 3D ^{13}C-filtered,^{13}C-methyl-separated NOESY (red, only ^{1}H-^{12}C to ^{1}H-^{13}C) and a 3D ^{13}C-edited NOESY (blue, any ^{1}H to ^{1}H-^{13}C) spectrum highlighting NOEs from the HD1 methyl group of I124. Some weak peaks in the ^{13}C-edited NOESY are intramolecular but are not observed in the ^{13}C-filtered,^{13}C-methyl-separated NOESY spectrum. **(b)** Amino acids in the vicinity of I124. Intramolecular NOEs are shown as dashed lines from blue amino acids. Likewise, intermolecular NOEs are shown as dashed lines from red amino acids.

a

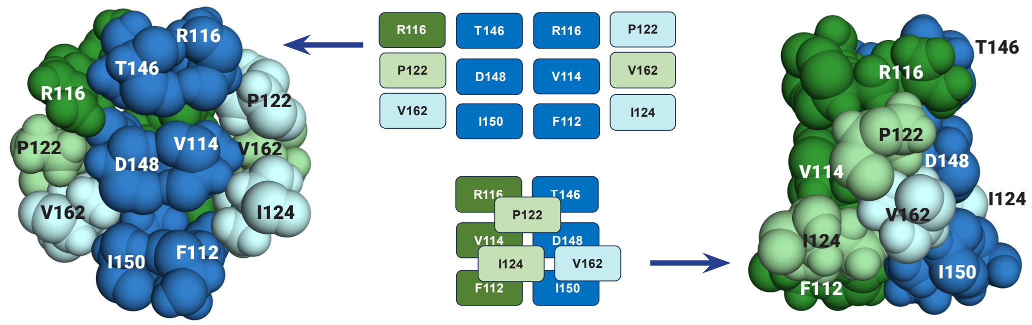

b

Residue	ASA (Å^2)	BSA (Å^2)	BSA/ASA (%)
110 ASN	97.6	0.0	0
111 GLN	83.1	0.0	0
112 PHE	79.2	44.8	57
113 LEU	0.2	0.0	0
114 VAL	32.0	31.1	97
115 VAL	0.0	0.0	0
116 ARG	144.3	101.3	70
117 HIS	53.5	0.0	0
118 PRO	71.6	0.0	0
119 GLY	85.9	0.0	0
120 LYS	155.2	6.2	4
121 THR	108.7	65.6	60
122 PRO	55.7	54.8	98
123 VAL	65.8	25.8	39
124 ILE	101.8	99.0	97
125 LYS	64.3	1.9	3
126 HIS	127.1	34.3	27
127 CYS	24.5	0.0	0
128 THR	126.5	0.0	0
129 GLY	58.5	0.0	0
130 ASP	83.5	0.0	0
131 LEU	31.1	0.0	0
132 GLU	49.0	0.0	0
133 GLU	90.2	0.0	0
134 PHE	30.9	0.0	0
135 LEU	0.2	0.0	0
136 ARG	102.9	0.0	0
137 GLN	82.6	0.0	0
138 LEU	24.0	0.0	0
139 ILE	64.3	0.0	0
140 GLU	112.3	0.0	0
141 GLN	149.3	0.0	0
142 ASP	25.1	0.0	0
143 PRO	103.0	0.0	0
144 LEU	135.8	0.0	0
145 VAL	15.0	0.0	0
146 THR	69.4	29.2	42
147 ILE	6.2	1.1	18
148 ASP	52.9	49.1	93
149 ILE	6.1	0.0	0
150 ILE	36.7	35.5	97
151 THR	46.7	0.0	0
152 HIS	133.9	2.4	2
153 ARG	122.3	0.0	0
154 TYR	212.4	0.0	0
155 TYR	185.7	0.0	0
156 GLY	36.0	0.0	0
157 VAL	109.8	56.1	51
158 GLY	77.2	14.4	19
159 GLY	28.9	0.0	0
160 GLN	94.4	75.8	80
161 TRP	73.3	19.4	27
162 VAL	72.6	72.2	99
163 GLN	20.6	3.9	19
164 ASP	90.3	61.6	68
165 ALA	3.8	0.0	0
166 GLY	34.5	0.0	0
167 GLU	102.9	19.5	19
168 TYR	20.4	0.0	0
169 LEU	21.1	0.0	0
170 HIS	125.5	0.0	0
171 MET	101.1	0.0	0
172 MET	73.8	0.0	0
173 SER	77.7	0.0	0
174 ASP	163.0	0.0	0

Figure S4 — Intermolecular interface analysis. **(a)** Two views of the interface with each protomer shaded green and blue. **(b)** Per residue PISA analysis (Krissinel & Henrick 2007; ASA; accessible surface area; BSA, buried surface area). Residues with a BSA/ASA ratio > 50% are shaded.

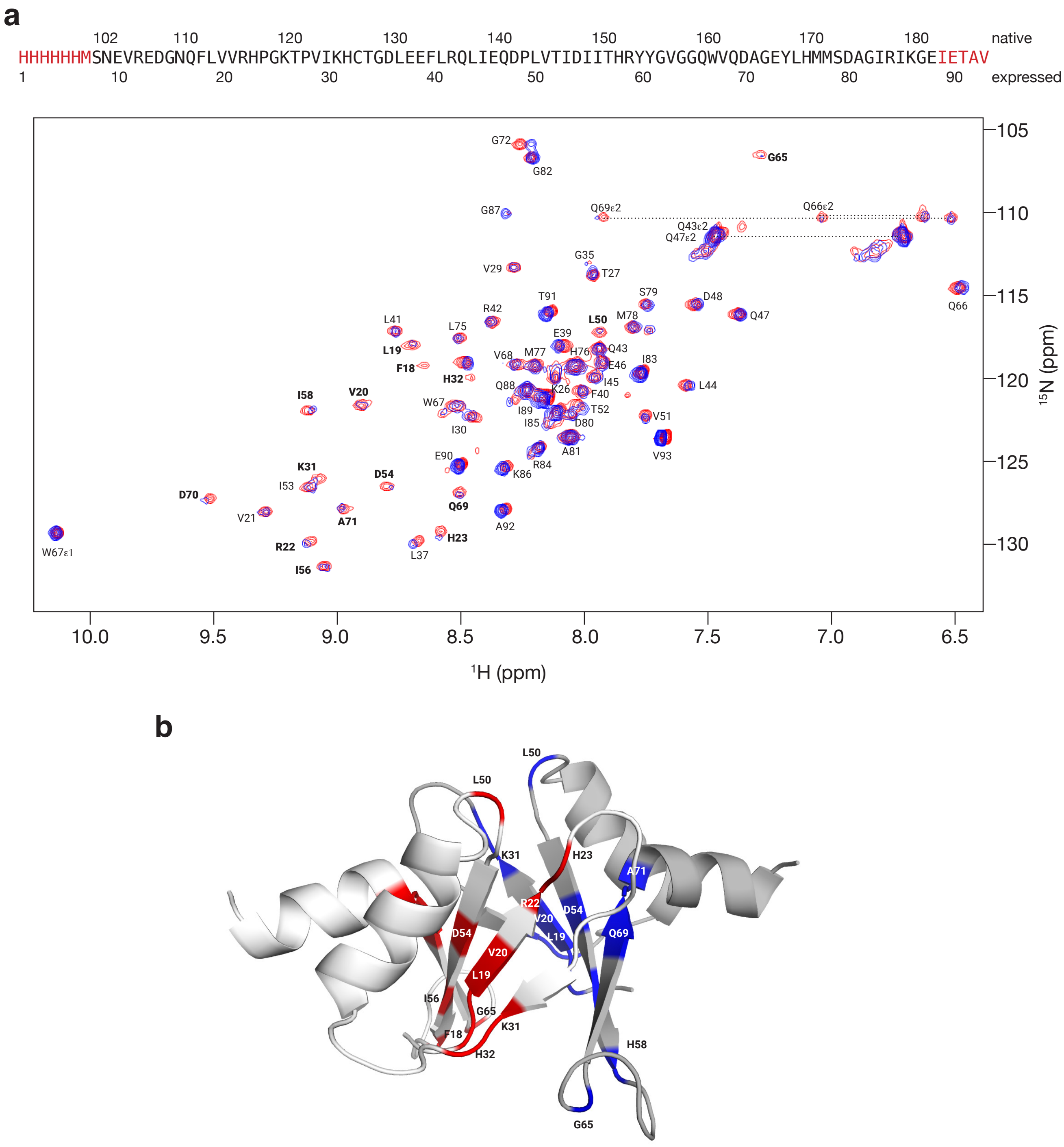

Figure S5 — (a) ^{15}N-HSQC spectra of the ^{15}N-Ea22 C-terminal domain (CTD) acquired at 298 K (blue) and 308 K (red). The protein concentration was 0.3 mM. Numbering follows the amino acids in the expressed protein. Non-native amino acids in the sequence introduced by cloning are indicated in red. **(b)** Backbone amide resonances that are weaker in the 25 ˚C spectrum than the 35 ˚C spectrum are mapped onto the Ea22 CTD structure and indicated in the spectrum by a label in boldface. Collectively, these resonances are located mainly at the dimer interface.

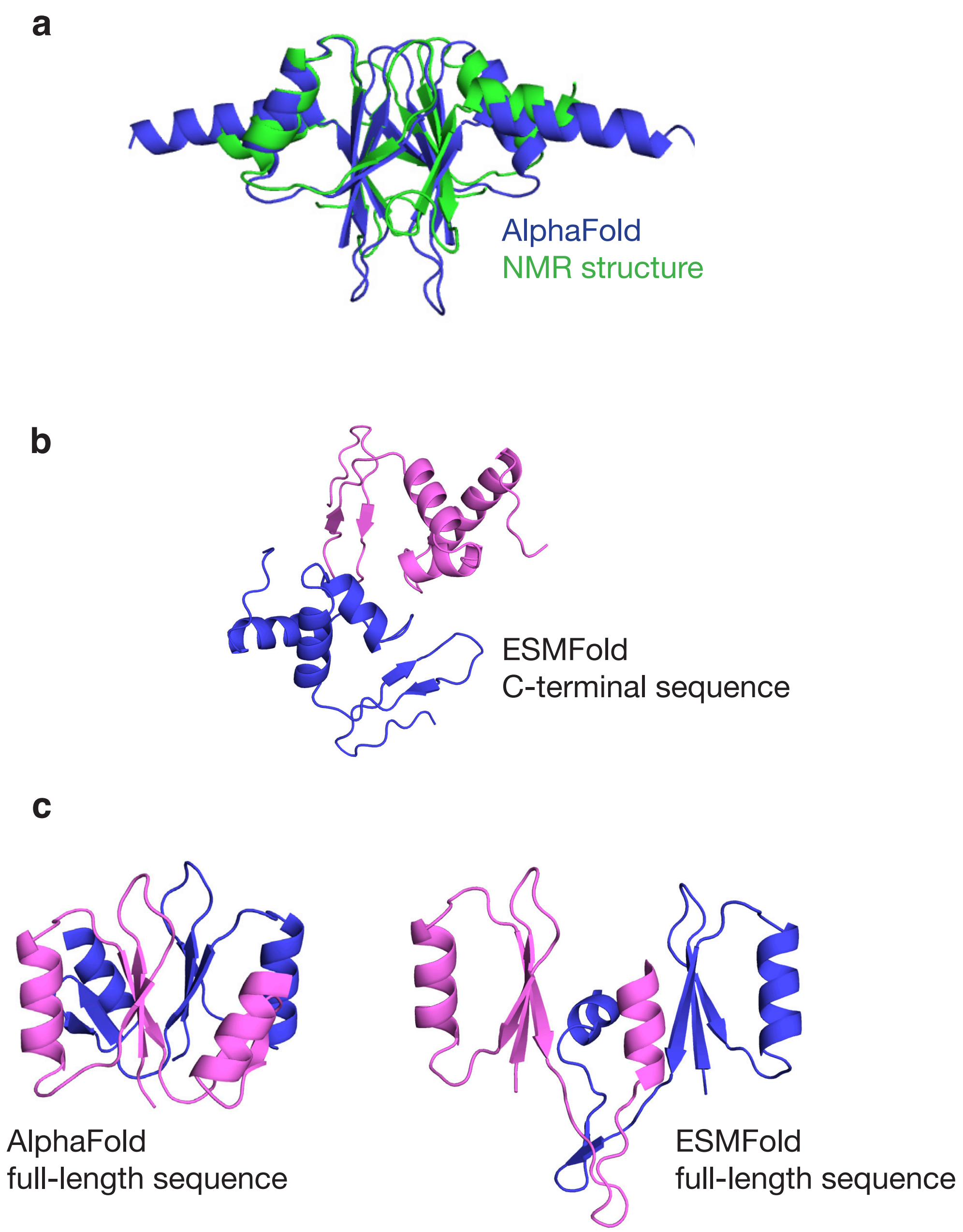

Figure S6 — Structural predictions of the λ Ea22 CTD dimer. **(a)** The AlphaFold prediction of the C-terminal sequence superimposed with the NMR solution structure. **(b)** A prediction by ESMFold of the C-terminal sequence. **(c)** A prediction by AlphaFold and ESMFold of the full-length Ea22 sequence.

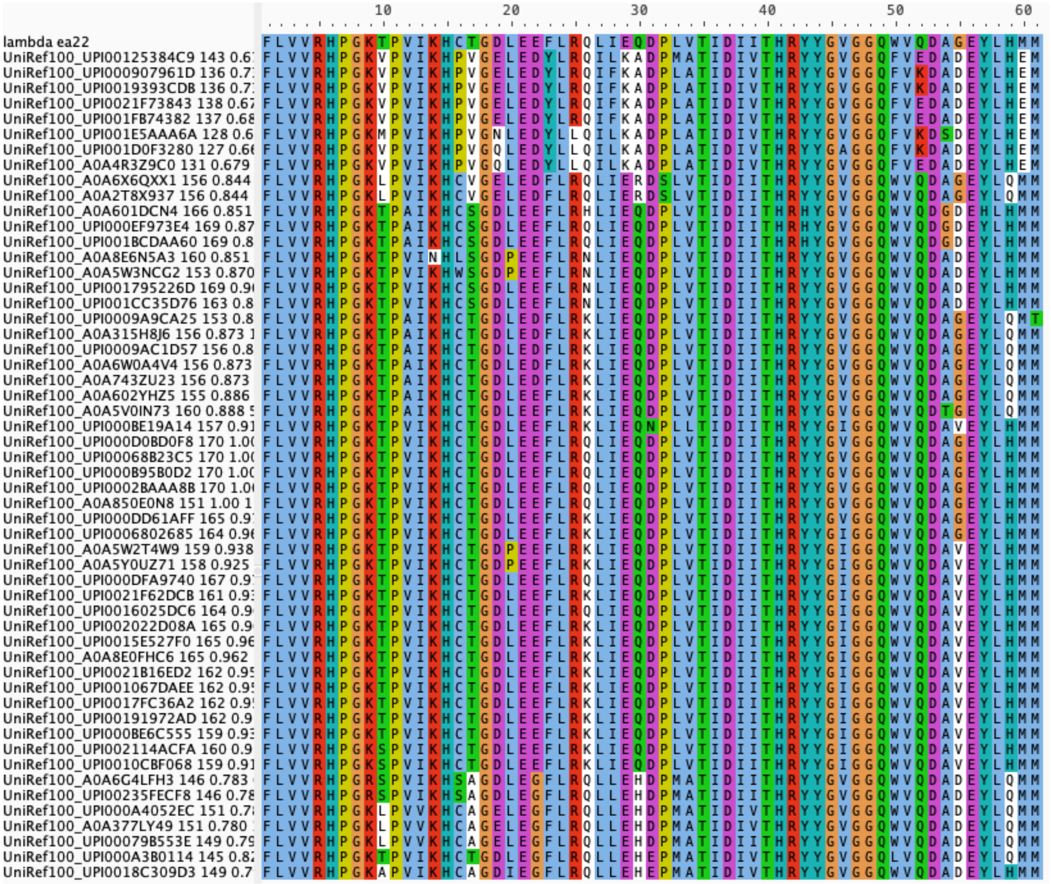

Figure S7 — Sequence alignment of the λ Ea22 carboxy terminal domain (CTD) and a set of close homologs identified in the UniRef100 database.

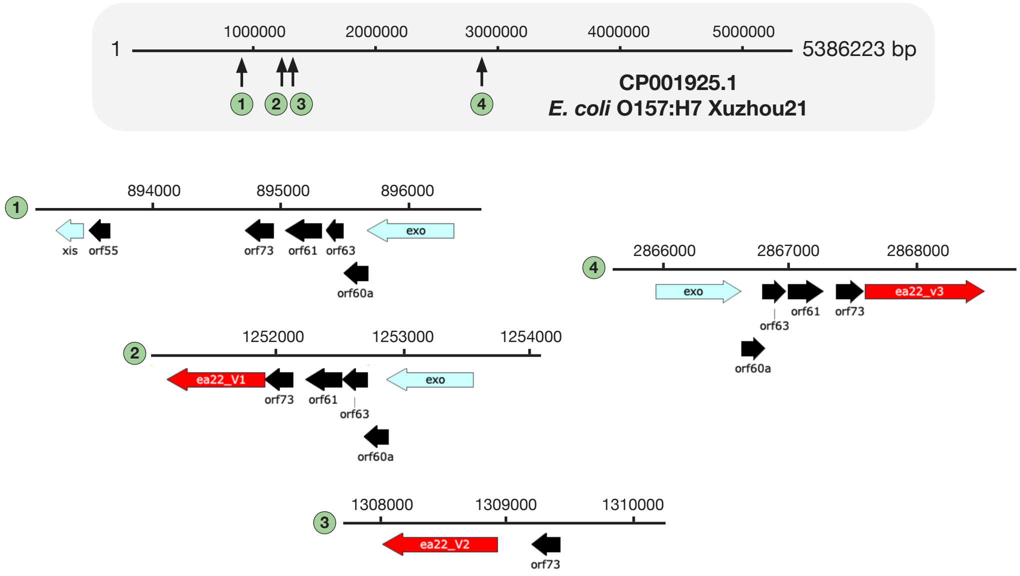

Figure S8 — Four *exo-xis* gene containing regions are present in the genome of *E. coli* O157:57 str. Xuzhou21.

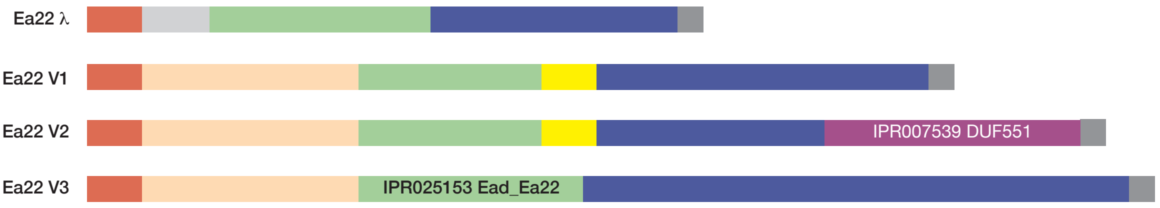


```
lambda      MSEINSQALREAAEQA
V1          MSEINYQALREKAEKA
V2          MSEINYQALREKAEKA
V3          MSEINYQALREKAEKA

lambda      MHDDWGFDADLFHELVTPSI--------------------------------------------
V1          TKGSYIVGHTSVNQHGNLTGVFVCQKWKGEPGGVIAECHVNCLIESDAQAYANAEFIAEANPAT
V2          TKGSYIVGHTSVNQHGNLTGVFVCQKWKGEPGGVIAECHVNCLIESDDQAYANAEFIAEANPAT
V3          TKGSYIVGHTSVNQHGNLTGVFVCQKWKGEPGGVIAECHVNCLVETDAQAYANAEFIAAFNPNV

lambda      VLELLDERERNQQYIKRRDQENEDIALTVGKLRVELETAKSKLNEQREYYEGVISDGSKRIAKLE
V1          VLELLDERERNQQYIKRRDQENEGIALTVGKLRVELEAAENNL-----------IDSECHVAELE
V2          VLALLDEQERNQQYIKRRDQENEEIALTVGKLRVELGAAENNL-----------IDSECHVAELE
V3          ALALLDERERNQQYIKRRDQENEDIALTVGKLRVELEAAKSKLNEQREYYEGVISDGSKHIAELE

lambda      SNEVREDGNQFLVVRHPGKTPVIKHCTGDLEEFLRQLIEQDPLVTIDIITHRYYGVGGQWVQDAGEYLHMMSD
V1          EALRDKLALLEASEKRNAKLQSENAYIRNRYKELDLLIGKNILVMQAAIIEWQATGDAKSGLAWIYNTLFGPG
V2          EALRDKQALLEASEKRIAELEAELVSQTYKLPHTQFEQIANLYEMQFDDGRTCAFHTDAQKAEQWLQACDGNR
V3          KQCAEWERKALSNFEECAAMAERIEEMQTKSAPDSFGIIGENIRTQDNRITSDPMFCVYQKREIVVDADYDYD

lambda      -----------------------------------------------------------------------------------------
V1          ELPDESEKDAQAYFNRKYAPIDEKLMALHKWFWEQSEAKRA------------------------------------------------
V2          VQEYVKLERLQNALSGNSPVTPDGWISCSERMPDTKTAVLVAVEFDRKGDWRMKWATYIPGHPDANDGWIIPGASWKPSHWMPLPEPP-
V3          RIVWVDEDGNEANKLQSRRLELLHENFREPPEKWRRVAVKDIDEFVTCCFTEQGCKDYLAVNGHNLRLPFIYVKSGFRNAEYIGIRNWL

lambda      AGIRIKGE
V1          AGIRIKGE
V2          QEVN-RG-
V3          AGIRIKGE
```

Figure S9 — Sequence comparison of λ Ea22 and three Ea22 variant prototypes determined from a survey of prophage and phage genomes. The V1 and V3 variants contain regions that are cataloged in the InterPro database.

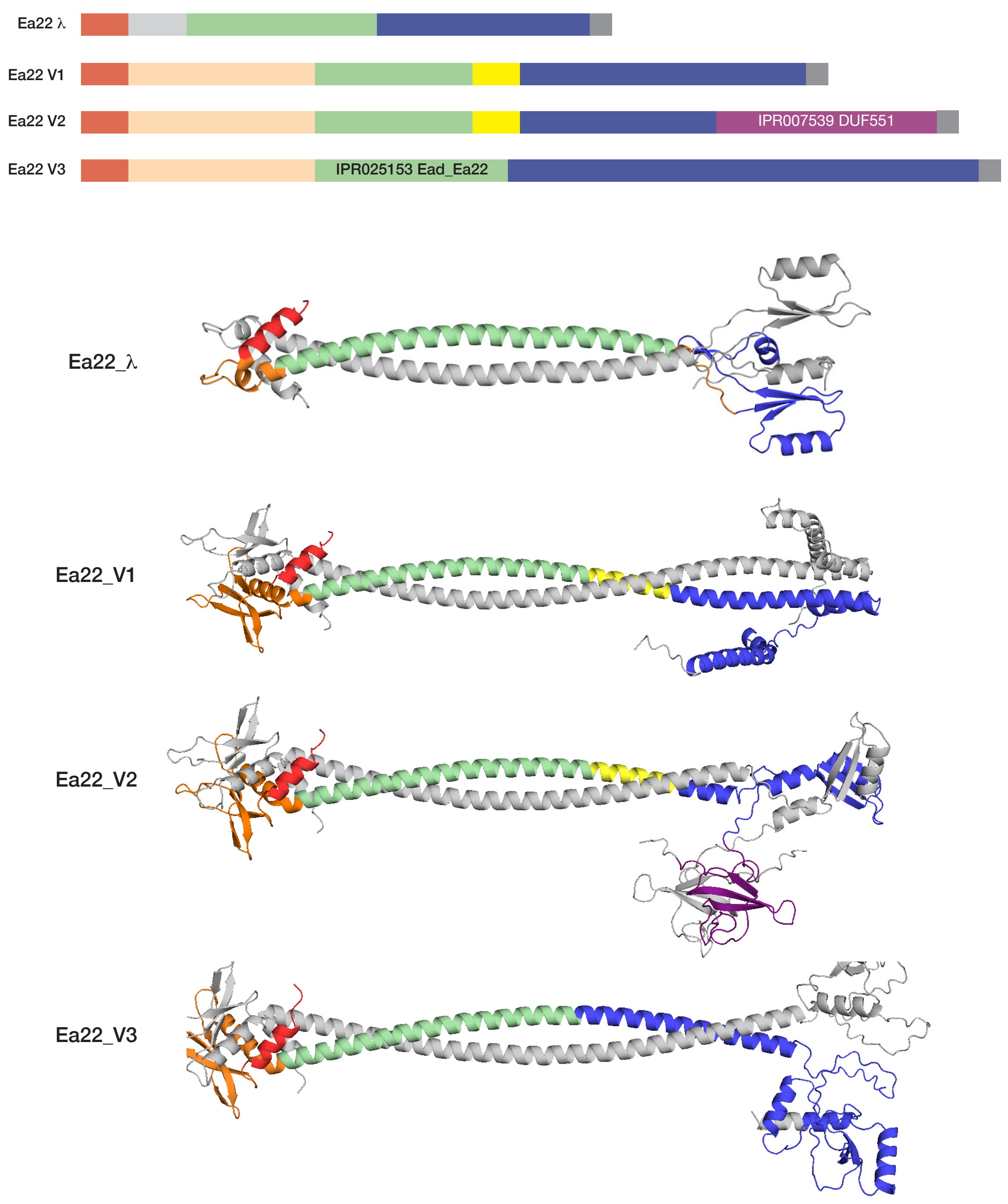

Figure S10 — ESMFold structure predictions of λ Ea22 and three Ea22 variant prototypes.
